# mRNA expression profiles obtained from microdissected pancreatic cancer cells can predict patient survival

**DOI:** 10.18632/oncotarget.20076

**Published:** 2017-08-03

**Authors:** Ana-Barbara García-García, M. Carmen Gómez-Mateo, Rebeca Hilario, Pilar Rentero-Garrido, Alvaro Martínez-Domenech, Veronica Gonzalez-Albert, Andres Cervantes, Pablo Marín-Garcia, Felipe Javier Chaves, Antonio Ferrández-Izquierdo, Luis Sabater

**Affiliations:** ^1^ CIBER of Diabetes and Associated Metabolic Diseases (CIBERDEM), Madrid, Spain; ^2^ Unidad de Genómica y Diagnóstico Genético. Fundación Investigación Clínico de Valencia, Instituto de Investigación Sanitaria Clínico de Valencia (INCLIVA), Valencia, Spain; ^3^ Department of Pathology, Faculty of Medicine and Odontology, University of Valencia and Clinical Hospital of Valencia, and Instituto de Investigación Sanitaria Clínico de Valencia (INCLIVA), Valencia, Spain; ^4^ Department of Surgery, University of Valencia, Hospital Clínico Universitario, Instituto de Investigación Sanitaria Clínico de Valencia (INCLIVA), Valencia, Spain; ^5^ Hematology and Medical Oncology Unit, Biomedical Research Institute INCLIVA and Department of Medicine, University of Valencia, Valencia, Spain; ^6^ Bioinformatics Unit. Fundación Investigación Clínico de Valencia, Instituto de Investigación Sanitaria Clínico de Valencia (INCLIVA), Valencia, Spain; ^7^ Current/Present address: Pathology Department, Hospital Universitario Donostia, San Sebastian, Spain

**Keywords:** pancreatic ductal adenocarcinoma, gene expression, patient survival, microdissected cells, regional lymph node metastases

## Abstract

**Background:**

Pancreatic ductal adenocarcinoma (PDAC) is one of the most devastating malignancies in developed countries because of its very poor prognosis and high mortality rates. By the time PDAC is usually diagnosed only 20-25% of patients are candidates for surgery, and the rate of survival for this cancer is low even when a patient with PDAC does undergo surgery. Lymph node invasion is an extremely bad prognosis factor for this disease.

**Methods:**

We analyzed the mRNA expression profile in 30 PDAC samples from patients with resectable local disease (stages I and II). Neoplastic cells were isolated by laser-microdissection in order to avoid sample ‘contamination’ by non-tumor cells. Due to important differences in the prognoses of PDAC patients with and without lymph node involvement (stage IIB and stages I-IIA, respectively), we also analyzed the association between the mRNA expression profiles from these groups of patients and their survival.

**Results:**

We identified expression profiles associated with patient survival in the whole patient cohort and in each group (stage IIB samples or stage I-IIA samples). Our results indicate that survival-associated genes are different in the groups with and without affected lymph nodes. Survival curves indicate that these expression profiles can help physicians to improve the prognostic classification of patients based on these profiles.

## INTRODUCTION

Pancreatic ductal adenocarcinoma (PDAC) is currently the fourth leading cause of cancer-related death in developed countries, and has a considerable economic and social impact [1]. Despite the availability of many treatment options, the prognosis for patients with PDAC remains very poor, with a 5-year survival rate of less than 3-4%. This type of tumor is usually diagnosed at a late stage (because symptoms do not usually present until the cancer is advanced), and this is directly related to the bad prognosis for this disease [2]. PDAC tends to rapidly invade surrounding structures and organs, metastasize early, and be highly resistant to both chemo- and radiation therapies.

Currently, surgery remains the only curative treatment for pancreatic cancer, but only 10-20% of patients are candidates for surgery at the time of diagnosis [3]. Even when patients undergo radical resection, only 20% of them remain alive after 5 years [4].

Unfortunately, there are currently no screening tests nor any useful biomarkers available for early PDAC detection which would allow pancreatic adenocarcinoma to be distinguished from other inflammatory pancreatic diseases like chronic pancreatitis, or which can be used to evaluate treatment responses or relapses in follow-up examinations [2].

The most commonly used marker for clinical diagnosis and to assess the effects of treatments is CA19-9. However, its sensitivity and specificity is not very high and its levels in serum can also be significantly increased in pancreas and biliary tract inflammatory diseases. This means that CA19-9 is not useful for predicting patient responses or prognoses [5]. Although many other approaches have been taken to find pancreatic cancer biomarkers, the clinical utility of these biomarkers remains to be determined, and many of these studies are still in their early phases. Genome analysis has shown that PDAC tumors contain a wide spectrum of mutations, however, only a few mutations are detected in most tumors (for instance those in the *KRAS* gene or loss/inactivation of known tumor suppressor genes, including *TP53* or *SMAD4*) [6]. Moreover, follow-up work comparing patient-matched primary PDAC tumors and subsequent metastases revealed the acquisition of further mutations in these metastases [7].

Enriching our knowledge of genes related to PDAC pathogenesis might allow us to develop tests to perform prognostic analyses and/or to identify new biomarkers or potential targets for therapy. One strategy is to analyze the transcriptome profile to try to detect genes with altered expression profiles in tumor cells and to identify any association they may have with survival time. There are specific problems for gene expression studies in pancreatic cancer because PDAC neoplastic cells often represent only a minor part of the tumoral-mass cell population, while dense desmoplastic stromal cells are the predominant component. Some different methods have been used to bypass these problems, including the use of pancreatic cancer cell lines [8], comparing a mixture of RNAs from pancreatitis and non-tumoral pancreas to pancreatic tumor cell RNA, and cancer cell enrichment by aspiration [9]. However, these approaches have their own limitations. Although several different studies have tried to detect a specific mRNA profile in different tumor stages, cell types, or patient survival groups, no definite prognostic signatures for patient survival have so far been identified [10, 11].

Laser-capture microdissection, which was first described several years ago [12], allows neoplastic epithelial cells to be isolated from non-tumoral cells, thus allowing the specific analysis of mRNAs from cancer cells while avoiding ‘contaminating’ the mixture with mRNA from non-cancerous cells. This method helps to solve some of the aforementioned problems regarding the study of gene expression in PDAC samples.

The aim the work we describe here was to analyze the association between mRNA profiles and patient survival. In order to reduce any interference from genes expressed in non-cancerous cells present in the tumor samples we used, we analyzed mRNA levels specifically in pancreatic ductal tumor cells which we selected by microdissecting samples from PDAC patients.

## RESULTS

After excluding cases with inadequate material (mixed histology, scant neoplastic pancreatic ductal material, etc.), we obtained enough RNA from the microdissected cells in 30 patient samples, which we then analyzed by microarray. These 30 patients had been followed-up for 20.75 ± 18.4 months in our oncology clinic; the patient and tumor characteristics are shown in Table 1.

**Table 1:** Clinical characteristics of the patients included in the study (whole group and subgroups)

	Whole group (stages I-II)	Subgroup A (stages I-IIA)	Subgroup B (stage IIB)
Age (years)	65 ± 8	66 ± 9	65 ± 7
Gender (male/female)	18/12	8/6	10/6
Follow-up (months)	31.97 ± 33.75	38.01 ± 41.96	28.31 ± 24.14
Survival (> 24 months / < 24 months, number)	13/17	7/7	6/10
Tumor length (cm)	3.15 ± 1.46	2.96 ± 1.66	3.27 ± 1.25
Lymph node ratio	0.15 ± 0.15	--	0.20 ± 0.16
Positive adenopathies	1.35 ± 2.10	--	2.94 ± 2.57
Lymphovascular invasion (yes/no)	18/12	7/7	11/5
Perineural invasion (yes/no)	19/11	8/6	11/5
Stage (number)	IA (3), IB (7), IIA(4), IIB (16)	IA (3), IB(7), IIA(4)	IIB (16)

Age, follow-up, tumor length, lymph node ratio, and positive adenopathies are indicated as mean ± standard deviation. Lymph node ratio: ratio of the number of metastatic lymph nodes to the number of removed lymph nodes.

### Analysis of the association between gene expression and survival

**Figure 1:**
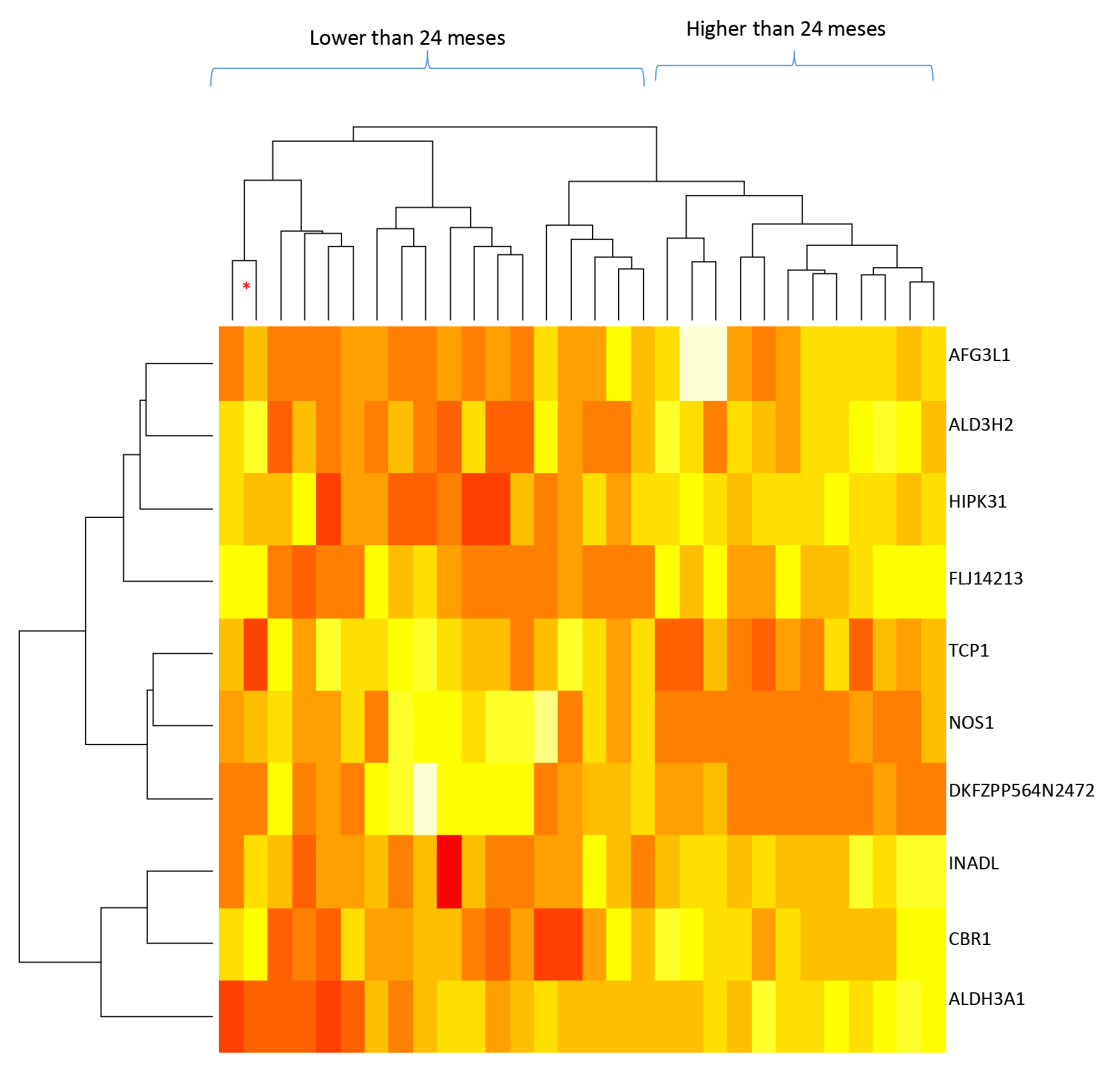
Dendrogram of mRNA levels and patient survival times for the whole sample (30 samples; stages IA to IIB) The genes included in this analysis are indicated on the right; red: lower mRNA levels, white: higher mRNA levels. The dendrogram at the top indicates the similarities between the samples and the one on the left indicates the similarity of the gene expression patterns. ^*^ indicates that the sample does not correspond to the dendrogram group it has been included in.

We analyzed mRNA expression levels in relation to patient survival (more or less than 24 months) which allowed us to identify 10 genes (Table 2) with altered mRNA levels in pancreatic ductal tumor cells compared to normal pancreatic cells (p < 0.001), although their association was not significant after Benjamini–Hochberg adjustment [13]. A dendrogram analysis of mRNA profiles (Figure 1) allowed us to correctly cluster all the samples into short (< 24 months) and long-term (> 24 months) survival groups, with one exception. This dendrogram also indicated that gene expression varied greatly between each patient and both survival groups.

**Table 2:** Genes with different levels of mRNA expression between shorter and longer survival-time groups

Gene	LogFC	P Value
FLJ14213	−1.761	0.00006
NOS1	1.078	0.00061
TCP1	0.759	0.00070
DKFZP564N2472	1.388	0.00086
INADL	−0.936	0.00087
CBR1	−0.930	0.00088
AFG3L1	−1.192	0.00088
ALDH3A1	−1.864	0.00089
HIPK3	−1.051	0.00093
ALDH3A2	−0.871	0.00098

A LogFC (logarithm of fold change) > 0 indicates a lower mRNA level in the longer survival-time group.

We decided to perform further analyses by classifying samples into two groups, depending on the presence of regional lymph node metastases: group A (without affected lymph nodes, stages IA to IIA), and group B (those with affected lymph nodes, only stage IIB). Group A included 14 samples (7 whose survival was less than 24 months); group B included 16 samples (10 whose survival was less than 24 months), see Table 1.

**Figure 2:**
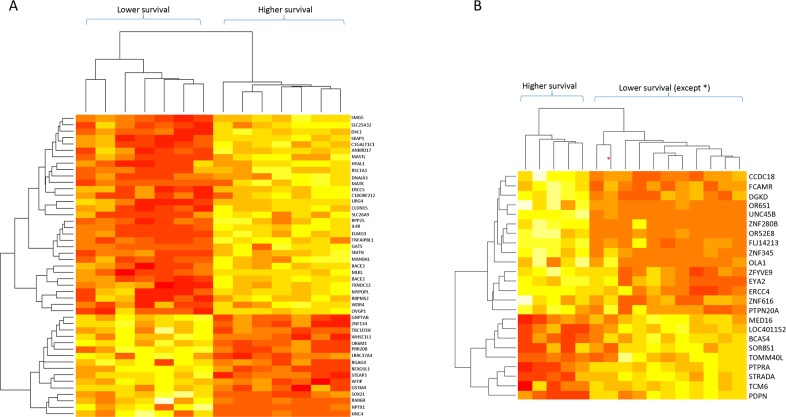
Dendrograms of mRNA levels and patient survival times for patient subgroups with stages I-IIA and IIB tumors **(A)** Stage I to IIA tumors (14 samples). **(B)** Stage IIB tumors (16 samples). The genes included in these analyses are indicated on the right; red: lower mRNA levels, white: higher mRNA levels.

Analysis of group A identified 47 genes whose differential expression was associated with patient survival (p < 0.001; Table 3). Figure 2A shows the results of the clustering analysis: samples with long and short survival times could be distinguished by the mRNA expression profile of these 47 genes. This figure also indicates that 16 genes were upregulated and 31 downregulated in patients with lower survival times. Group B analysis identified 24 genes whose differential expression was associated with patient survival (p < 0.001; Table 4). Clustering analysis of this group (Figure 2B) also showed that samples with long and short survival times could be distinguished by the expression profile of these 24 mRNAs (except for one sample). Nine of these genes were upregulated and 15 were downregulated in shorter survival-time patients. In contrast to the dendrogram from the whole group, the dendrograms shown in Figure 2A and 2B also show that there was higher gene-expression homogeneity in samples from stages I-IIA (Group A) or stage 2B (Group B).

**Table 3:** Group A: Genes with different levels of mRNA expression between shorter and longer survival-time groups

Gene	LogFC	P Value	Gene	LogFC	P Value
REXO1L1	3.392	0.000002	STEAP3	1.341	0.00040
OR6M1	2.436	0.000004	C1GALT1C1	−1.515	0.00048
MLKL	−2.549	0.00001	WTIP	1.779	0.00048
OVGP1	−2.810	0.00002	BACE2	−2.134	0.00051
SKAP1	−2.270	0.00003	WDR4	−1.755	0.00053
ELMO3	−1.815	0.00003	SMTN	−1.605	0.00058
BACE2	−1.821	0.00003	LRRC37A4	1.816	0.00061
MATK	−2.766	0.00004	ERCC5	−2.045	0.00069
DVL1	−1.896	0.00006	HYAL1	−2.099	0.00069
SMG5	−1.863	0.00007	TBC1D3H	1.774	0.00069
TNFAIP8L1	−1.652	0.00011	GSTM4	1.882	0.00069
IL4R	−1.306	0.00011	ANKRD17	−1.862	0.00072
SLC26A9	−2.709	0.00013	MANBAL	−1.731	0.00074
CLDN15	−2.836	0.00014	SLC25A32	−1.632	0.00076
MYPOP	−3.167	0.00021	C1ORF212	−1.990	0.00078
WHSC1L1	1.817	0.00025	URG4	−2.589	0.00079
GNPTAB	1.558	0.00027	RAB6B	1.701	0.00081
ZNF134	1.721	0.00029	TXNDC12	−2.052	0.00082
PRR20B	2.223	0.00030	RPP25	−1.236	0.00083
NPTX1	2.033	0.00030	RBPMS2	−2.745	0.00085
GATS	−1.687	0.00034	RGAG4	2.217	0.00091
DNAJA3	−2.083	0.00035	SOX21	1.665	0.00093
RSC1A1	−2.115	0.00037	HCN4	2.413	0.00099
MASTL	−2.506	0.00040			

A LogFC (logarithm of fold change) > 0 indicates a lower mRNA level in the longer survival-time group.

**Table 4:** Group B: Genes with different levels of mRNA expression between shorter and longer survival-time groups

Gene	LogFC	P Value
ZNF345	−2.708	0.000001
STRADA	2.334	0.000087
OR52E8	−2.132	0.000091
UNC45B	−1.461	0.000092
FLJ14213	−2.291	0.000149
FCAMR	−1.540	0.000175
MED16	1.606	0.000261
CCDC18	−1.580	0.000359
DGKD	−1.918	0.000363
PDPN	2.856	0.000387
ZFYVE9	−1.767	0.000395
TOMM40L	1.860	0.000412
OR6S1	−1.613	0.000423
OLA1	−1.690	0.000432
SORBS1	2.279	0.000534
PTPN20A	−2.262	0.000576
ERCC4	−2.034	0.000624
PTPRA	1.693	0.000626
ZNF280B	−1.861	0.000637
EYA2	−1.631	0.000688
ZNF616	−2.002	0.000734
BCAS4	2.081	0.000805
TMC6	1.904	0.000883
ZNF548	−1.577	0.000911

LogFC (logarithm of fold change) > 0 indicates a lower mRNA level in the longer survival-time group.

**Figure 3:**
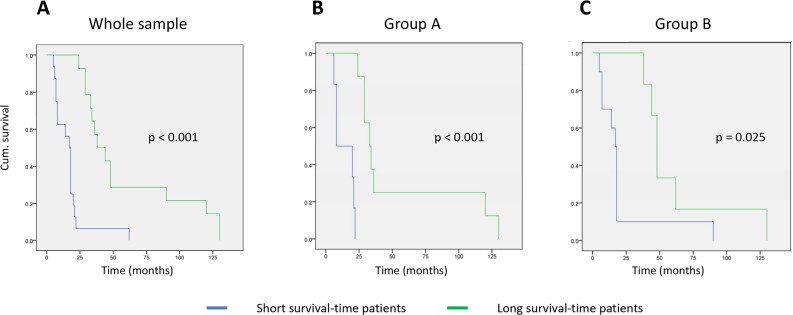
Kaplan–Meyer curves for patient survival based on the mRNA-profile classification **(A)** Whole sample group; **(B)** group A (stages I to IIA tumors); **(C)** group B (stage IIB tumors).

Finally, we analyzed the patient survival curves according to the mRNA profile classifications described above (Figure 3). In the whole group (30 samples), there was a significant difference between the short and long survival-time cohorts classified by these mRNA profiles (p < 0.001); the mean survival was 16.8 months (95% CI: 10.2-23.4 months) and 59.5 months (95% CI: 38.6-80.3 months), respectively. In group A, the survival curves were statistically different to those for the whole group (p < 0.001) with a mean survival time for each subgroup of 14.1 months (95% CI: 8.1-20.2 months) vs. 54.3 months (95% CI: 24.0-84.7 months), respectively. Finally, in group B the difference in survival was maintained between the curves (p = 0.025) and the median subgroup survival times were 21.2 months (95% CI: 5.8-36.5 months) and 61.7 months (95% CI: 34.1-89.2 months), respectively.

## DISCUSSION

To date, some studies on gene expression profiles in pancreatic cancer focusing on differential expression between normal and tumor tissues or cell lines have been published [14–18]. However, the findings of these studies are controversial and there is little concordance between the results. Data from these types of studies have been integrated into a meta-analysis which gave interesting results [19], as well as into the Pancreatic Expression Database [20] and Pancreatic Cancer Database (http://www.pancreaticcancerdatabase.org). These types of retrospective data integration analyses have helped to determine that differences in the results may be due to design biases such as tissue or sample selection, cancer cell enrichment procedures, the type of microarray used, or because of differences between tumors and their development, statistical limitations, etc.

Some studies have focused on detecting differences between primary tumors and metastases and have found different signatures related to prognosis or survival [10, 21]. Others have analyzed PDAC samples from patients with and without affected lymph nodes and have identified many genes that are differentially expressed between these groups [22, 23]. Donahue et al. (2012) [24] identified and analyzed the mRNA levels of 171 genes which were able to define two prognosis groups based on their probability of disease-free survival. However, variation in the study designs and the use of different methodologies between these studies (including the patient inclusion criteria, tumor type and stage, comparison of primary or metastatic tumors, purification methods, or microarray technologies used) have produced considerable differences in the results. Therefore, little progress has so far been made in the study of associations between mRNA profiles and survival [10, 24].

The presence of non-tumor cells in the samples (which are more abundant in PDAC compared to other cancer types), and the altered expression levels of a wide range of genes, can lead to distorted results [19, 25, 26]. In order to avoid this problem, we microdissected tumor cells and only extracted RNA from them. In addition, we only included tumors which were surgically resectable and without distal metastases (stages IA, IB, IIA, and IIB). We analyzed three separate cohorts: first, the entire sample group, second, patients with tumor stages IA to IIA (group A, without affected lymph nodes), and third, subjects with tumor stage IIB (group B, with affected lymph nodes).

Different genes were identified in each of the three cohorts, indicating that there is a lot of variability in the mRNA expression associated with survival in each group, which may help to explain the large amount of variability found in the group as a whole. Our results improve upon previously published data because we were able to analyze isolated tumor cells, and we took the sample cancer stage into consideration.

Some genes in the overall sample group could be related to tumor progression in association with patient survival; only *NOS1*, *TCP1*, and DKFZ*p564*N2472 had higher expression levels in patients with lower survival, while the remaining genes had lower expression levels in these patients. Some of the genes with differences in mRNA levels in the whole group (*FLJ14213*, *NOS1*, *TCP1*, *INADL*, *ALDH3A1*, and *ALDH3A2*) have been previously identified as having altered expression in different pancreatic tumor tissues or cells, according to the Pancreatic Expression Database [20] and the Pancreatic Cancer Database (http://www.pancreaticcancerdatabase.org). *HIPK3* has been related to apoptosis resistance [27], *CBR1* has been identified as a patient survival marker [24], *ALDH3A1* and *ALDH3A2* have been related to drug response [28], and *NOS1* has been related to pancreatic cancer risk [29].

Many of the genes identified in group A with different expression in the long-term survival group had also been previously identified as being altered in PDAC, according to the same aforementioned databases; these include: *SKAP1*, *ELMO3*, *BACE2*, *TNFAIP8L1*, *IL4R*, *SLC26A9*, *WHSC1L1*, *GNPTAB*, *ZNF134*, *NPTX1*, *GATS*, *MASTL*, *STEAP3*, *C1GALT1C1*, *WDR4*, *SMTN*, *HYAL1*, *GSTM4*, *ANKRD17*, *C1ORF212*, *RBPMS2*, *TXNDC12*, *RPP25*, *HCN4*, and *DVL1* [9, 17, 19, 24, 26, 30]. Interestingly, some genes have been implicated in the development of prognostic markers in other tumors, including: *MLKL* [31], *SKAP1* [32], *DVL1* [33], *DNAJA3* [34], *ERCC5* [35], *URG4* [36], and *RBPMS2* [37]. Additionally, *MATK* has been related to cell invasion [38] and *IL4R* expression in cancer cells seems to facilitate lymph node metastasis [39].

We identified many genes that are differentially expressed between the long and short survival-time groups in group B, some of which have previously been found to be altered in pancreatic cancer such as *ZNF345*, *ZNF280B*, *UNC45B*, *ZFYVE9*, *DGKD*, *PDPN*, *OLA1*, *SORBS1*, *PTPN20A*, *PTPRA*, *EYA2*, and *BCAS4* (according to the Pancreatic Expression and Pancreatic Cancer Databases). Other genes represent a genetic variation which correlates with increased pancreatic cancer risk (*ERCC4*) [40], have been related to pancreatic cancer development (*CCDC18* or *DGKD*) [41, 42], can predict the prognosis or risk for other cancers (*PDPN* and *TMC6*) [43, 44], or have been related to cell proliferation and migration (*EYA2*) [45]. It is interesting to note that these include three zinc finger genes (*ZNF345*, *ZNF280B*, and *ZNF616*) which may indicate the presence of an important alteration in gene regulation in PDAC.

Taken together these data indicate that there is a lot of variability among genes that are altered in, and/or related to, pancreatic tumor development, progression, and survival. Our results show that different gene expression profiles are associated with the survival of patients with tumors with or without local lymph node involvement. These profiles may be useful to help guide future decision making regarding treatment options for PDAC patients. However, it is important to note that here we have analyzed only a small sample and that only some of the genes obtained may be really involved in this classification. Our results should be confirmed in a wider population in future studies.

## MATERIALS AND METHODS

### Patients

For this study we selected patients with PDAC stages I and II who had undergone surgery for the disease between 1998 and 2010 and who had both a full clinical follow-up until death and sufficient and appropriate histological material for analysis. Overall survival was considered to be the time from PDAC diagnosis to death. The Ethics Committee at the *Hospital Clínico Universitario* in Valencia approved this work and all the patients gave their informed written consent for their samples to be included in the INCLIVA Biobank.

### Tissue samples

We collected the histological material (paraffin-embedded tumor tissue) from the INCLIVA Biobank. Histological sections from all the samples were reviewed by two pathologists to confirm the diagnosis and rule out any cases presenting a mixed-type histology. The most representative block was selected from each case. The paraffin blocks were cut into 6-μm sections on slides, deparaffinized, and stained with sterile hematoxylin and eosin in order to visualize the tumor cells (in RNase-free conditions at all stages of the process). Sections were then microdissected in an AS-LMD Laser Microdissection System (Leica Microsystems) to obtain a minimum of 10,000 cells per sample.

From the 44 patients we initially selected, four cases were discarded because of mixed-type histology, four cases had insufficient tumoral material for microdissection, and in three cases the paraffin blocks were not usable. We eventually processed 33 samples and obtained about 5 ng of total RNA from each sample using a High Pure FFPE RNA Micro kit (Roche). RNA from each sample was proportionally amplified with a Sensation TM RNA Amplification kit (Genisphere) to obtain sufficient RNA for a chip assay. After this process we obtained enough RNA for microarray analysis from 30 of the samples.

### Expression studies

We used human HT-12-v4 expression BeadChips for Whole Genome DASL assays (Illumina). Samples were loaded randomly into an Illumina HiScan system to avoid bias in the analysis of the different groups, and we followed the manufacturer's “Whole Genome DASL Assay” protocol. Raw data was obtained using the Genome Studio (version 2011.01) program and the Gene Expression module (v.1.9.0) from Illumina, without normalizing. A quality analysis screen was performed using the Illumina Genome Studio software (v.2011.1). The “Average Signal”, “Bead Standard Deviation”, “Average Number of Beads”, and “Detection p Value” columns were exported, as recommended by the BeadArray package used in the analysis.

Data analysis was performed with R with the Bioconductor module (R_2.14.0 and Biobase_2.14.0), using the BeadArray package for quality control and normalization (Beadarray_2.4.1). Samples were normalized using the QC Spline method. Other packages used were Limma (statistical analysis) and genefilter (sample filtering). Probability p values were adjusted using Benjamini–Hochberg correction [15]. Microarray data were submitted to GEO (accession no. GSE84219).

### Statistical analysis

For descriptive analysis, patient data were analyzed using SPSS (v.22) software and the data was expressed as the mean ± the standard deviation. Kaplan–Meier survival curves based on classifications obtained for the mRNA profiles were also created using SPSS (v.22) software.

## References

[1] Ferlay J, Shin HR, Bray F, Forman D, Mathers C, Parkin DM (2010). Estimates of worldwide burden of cancer in 2008: GLOBOCAN 2008. Int J Cancer.

[2] Stathis A, Moore MJ (2010). Advanced pancreatic carcinoma: current treatment and future challenges. Nat Rev Clin Oncol.

[3] Yeo CJ, Cameron JL, Sohn TA, Coleman J, Sauter PK, Hruban RH, Pitt HA, Lillemoe KD (1999). Pancreaticoduodenectomy with or without extended retroperitoneal lymphadenectomy for periampullary adenocarcinoma: comparison of morbidity and mortality and short-term outcome. Ann Surg.

[4] Hawes RH, Xiong Q, Waxman I, Chang KJ, Evans DB, Abbruzzese JL (2000). A multispecialty approach to the diagnosis and management of pancreaticcancer. Am J Gastroenterol.

[5] Ghaneh P, Costello E, Neoptolemos JP (2007). Biology and management of pancreatic cancer. Gut.

[6] Waddell N, Pajic M, Patch AM, Chang DK, Kassahn KS, Bailey P, Johns AL, Miller D, Nones K, Quek K, Quinn MC, Robertson AJ, Fadlullah MZ (2015). Whole genomes redefine the mutational landscape of pancreatic cancer. Nature.

[7] Campbell PJ, Yachida S, Mudie LJ, Stephens PJ, Pleasance ED, Stebbings LA, Morsberger LA, Latimer C, McLaren S, Lin ML, McBride DJ, Varela I, Nik-Zainal SA (2010). The patterns and dynamics of genomic instability in metastatic pancreatic cancer. Nature.

[8] Gress TM, Müller-Pillasch F, Geng M, Zimmerhackl F, Zehetner G, Friess H, Büchler M, Adler G, Lehrach H (1996). A pancreatic cancer-specific expression profile. Oncogene.

[9] Crnogorac-Jurcevic T, Efthimiou E, Capelli P, Blaveri E, Baron A, Terris B, Jones M, Tyson K, Bassi C, Scarpa A, Lemoine NR (2001). Gene expression profiles of pancreatic cancer and stromal desmoplasia. Oncogene.

[10] Stratford JK, Bentrem DJ, Anderson JM, Fan C, Volmar KA, Marron JS, Routh ED, Caskey LS, Samuel JC, Der CJ, Thorne LB, Calvo BF, Kim HJ (2010). A six-gene signature predicts survival of patients with localized pancreatic ductal adenocarcinoma. PLoS Med.

[11] Collisson EA, Sadanandam A, Olson P, Gibb WJ, Truitt M, Gu S, Cooc J, Weinkle J, Kim GE, Jakkula L, Feiler HS, Ko AH, Olshen AB (2011). Subtypes of pancreatic ductal adenocarcinoma and their differing responses to therapy. Nat Med.

[12] Emmert-Buck MR, Bonner RF, Smith PD, Chuaqui RF, Zhuang Z, Goldstein SR, Weiss RA, Liotta LA (1996). Laser capture microdissection. Science.

[13] Benjamini Y, Hochberg Y (1995). Controlling the false discovery rate: a practical and powerful approach to multiple testing. J Royal Stat Soc.

[14] Han H, Bearss DJ, Browne LW, Calaluce R, Nagle RB, Von Hoff DD (2002). Identification of differentially expressed genes in pancreatic cancer cells using cDNA microarray. Cancer Res.

[15] Iacobuzio-Donahue CA, Ashfaq R, Maitra A, Adsay NV, Shen-Ong GL, Berg K, Hollingsworth MA, Cameron JL, Yeo CJ, Kern SE, Goggins M, Hruban RH (2003). Highly expressed genes in pancreatic ductal adenocarcinomas: a comprehensive characterization and comparison of the transcription profiles obtained from three major technologies. Cancer Res.

[16] Tan ZJ, Hu XG, Cao GS, Tang Y (2003). Analysis of gene expression profile of pancreatic carcinoma using cDNA microarray. World J Gastroenterol.

[17] Nakamura T, Furukawa Y, Nakagawa H, Tsunoda T, Ohigashi H, Murata K, Ishikawa O, Ohgaki K, Kashimura N, Miyamoto M, Hirano S, Kondo S, Katoh H (2004). Genome-wide cDNA microarray analysis of gene expression profiles in pancreatic cancers using populations of tumor cells and normal ductal epithelial cells selected for purity by laser microdissection. Oncogene.

[18] Grützmann R, Boriss H, Ammerpohl O, Lüttges J, Kalthoff H, Schackert HK, Klöppel G, Saeger HD, Pilarsky C (2005). Meta-analysis of microarray data on pancreatic cancer defines a set of commonly dysregulatedgenes. Oncogene.

[19] Gadaleta E, Cutts RJ, Kelly GP, Crnogorac-Jurcevic T, Kocher HM, Lemoine NR, Chelala C (2011). A global insight into a cancer transcriptional space using pancreatic data: importance, findings and flaws. Nucleic Acids Res.

[20] Dayem Ullah AZ, Cutts RJ, Ghetia M, Gadaleta E, Hahn SA, Crnogorac-Jurcevic T, Lemoine NR, Chelala C (2014). The pancreatic expression database: recent extensions and updates. Nucleic Acids Res.

[21] Ramaswamy S, Ross KN, Lander ES, Golub TR (2003). A molecular signature of metastasis in primary solid tumors. Nat Genet.

[22] Kim HN, Choi DW, Lee KT, Lee JK, Heo JS, Choi SH, Paik SW, Rhee JC, Lowe AW (2007). Gene expression profiling in lymph node-positive and lymph node-negative pancreatic cancer. Pancreas.

[23] Hirono S, Yamaue H, Hoshikawa Y, Ina S, Tani M, Kawai M, Ushijima M, Matsuura M, Saiki Y, Saiura A, Yamamoto J, Miki Y, Noda T (2010). Molecular markers associated with lymph node metastasis in pancreatic ductal adenocarcinoma by genome-wide expression profiling. Cancer Sci.

[24] Donahue TR, Tran LM, Hill R, Li Y, Kovochich A, Calvopina JH, Patel SG, Wu N, Hindoyan A, Farrell JJ, Li X, Dawson DW, Wu H (2012). Integrative survival-based molecular profiling of human pancreatic cancer. Clin Cancer Res.

[25] Badea L, Herlea V, Dima SO, Dumitrascu T, Popescu I (2008). Combined gene expression analysis of whole-tissue and microdissected pancreatic ductal adenocarcinoma identifies genes specifically overexpressed in tumor epithelia. Hepatogastroenterology.

[26] Fukushima N, Koopmann J, Sato N, Prasad N, Carvalho R, Leach SD, Hruban RH, Goggins M (2005). Gene expression alterations in the non-neoplastic parenchyma adjacent to infiltrating pancreatic ductal adenocarcinoma. Mod Pathol.

[27] Curtin JF, Cotter TG (2004). JNK regulates HIPK3 expression and promotes resistance to Fas-mediated apoptosis in DU 145 prostate carcinoma cells. J Biol Chem.

[28] Ma I, Allan AL (2011). The role of human aldehyde dehydrogenase in normal and cancer stem cells. Stem Cell Rev.

[29] Reid-Lombardo KM, Fridley BL, Bamlet WR, Cunningham JM, Sarr MG, Petersen GM (2011). Inflammation-related gene variants as risk factors for pancreatic cancer. Cancer Epidemiol Biomarkers Prev.

[30] Nakamura T, Kuwai T, Kitadai Y, Sasaki T, Fan D, Coombes KR, Kim SJ, Fidler IJ (2007). Zonal heterogeneity for gene expression in human pancreatic carcinoma. Cancer Res.

[31] He L, Peng K, Liu Y, Xiong J, Zhu FF (2013). Low expression of mixed lineage kinase domain-like protein is associated with poor prognosis in ovarian cancer patients. Onco Targets Ther.

[32] Goode EL, Chenevix-Trench G, Song H, Ramus SJ, Notaridou M, Lawrenson K, Widschwendter M, Vierkant RA, Larson MC, Kjaer SK, Birrer MJ, Berchuck A, Schildkraut J (2010). A genome-wide association study identifies susceptibility loci for ovarian cancer at 2q31 and 8q24. Nat Genet.

[33] Huang MY, Yen LC, Liu HC, Liu PP, Chung FY, Wang TN, Wang JY, Lin SR (2013). Significant overexpression of DVL1 in Taiwanese colorectal cancer patients with liver metastasis. Int J Mol Sci.

[34] Jan CI, Yu CC, Hung MC, Harn HJ, Nieh S, Lee HS, Lou MA, Wu YC, Chen CY, Huang CY, Chen FN, Lo JF (2011). Tid1, CHIP and ErbB2 interactions and their prognostic implications for breast cancer patients. J Pathol.

[35] Schrama D, Scherer D, Schneider M, Zapatka M, Bröcker EB, Schadendorf D, Ugurel S, Kumar R, Becker JC (2011). ERCC5 p.Asp1104His and ERCC2 p.Lys751Gln polymorphisms are independent prognostic factors for the clinical course of melanoma. J Invest Dermatol.

[36] Li W, Zhou N (2012). URG4 upregulation is associated with tumor growth and poor survival in epithelial ovarian cancer. Arch Gynecol Obstet.

[37] Hapkova I, Skarda J, Rouleau C, Thys A, Notarnicola C, Janikova M, Bernex F, Rypka M, Vanderwinden JM, Faure S, Vesely J, de Santa Barbara P (2013). High expression of the RNA-binding protein RBPMS2 in gastrointestinal stromal tumors. Exp Mol Pathol.

[38] Fu Y, Zagozdzon R, Avraham R, Avraham HK (2006). CHK negatively regulates Lyn kinase and suppresses pancreatic cancer cell invasion. Int J Oncol.

[39] Formentini A, Prokopchuk O, Sträter J, Kleeff J, Grochola LF, Leder G, Henne-Bruns D, Korc M, Kornmann M (2009). Interleukin-13 exerts autocrine growth-promoting effects on human pancreatic cancer, and its expression correlates with a propensity for lymph node metastases. Int J Colorectal Dis.

[40] Hocevar BA, Kamendulis LM, Pu X, Perkins SM, Wang ZY, Johnston EL, DeWitt JM, Li L, Loehrer PJ, Klaunig JE, Chiorean EG (2014). Contribution of environment and genetics to pancreatic cancer susceptibility. PLoS One.

[41] Gylfe AE, Katainen R, Kondelin J, Tanskanen T, Cajuso T, Hänninen U, Taipale J, Taipale M, Renkonen-Sinisalo L, Järvinen H, Mecklin JP, Kilpivaara O, Pitkänen E (2013). Eleven candidate susceptibility genes for common familial colorectal cancer. PLoS Genet.

[42] Whitworth H, Bhadel S, Ivey M, Conaway M, Spencer A, Hernan R, Holemon H, Gioeli D (2012). Identification of kinases regulating prostate cancer cell growth using an RNAi phenotypic screen. PLoS One.

[43] Choi SY, Sung R, Lee SJ, Lee TG, Kim N, Yoon SM, Lee EJ, Chae HB, Youn SJ, Park SM (2013). Podoplanin, α-smooth muscle actin or S100A4 expressing cancer-associated fibroblasts are associated with different prognosis in colorectal cancers. J Korean Med Sci.

[44] Castro FA, Ivansson EL, Schmitt M, Juko-Pecirep I, Kjellberg L, Hildesheim A, Gyllensten UB, Pawlita M (2012). Contribution of TMC6 and TMC8 (EVER1 and EVER2) variants to cervical cancer susceptibility. Int J Cancer.

[45] Fu J, Xu X, Kang L, Zhou L, Wang S, Lu J, Cheng L, Fan Z, Yuan B, Tian P, Zheng X, Yu C, Ye Q (2014). miR-30a suppresses breast cancer cell proliferation and migration by targeting Eya2. Biochem Biophys Res Commun.

